# A comparative patient-level prediction study in OMOP CDM: applicative potential and insights from synthetic data

**DOI:** 10.1038/s41598-024-52723-y

**Published:** 2024-01-27

**Authors:** Najia Ahmadi, Quang Vu Nguyen, Martin Sedlmayr, Markus Wolfien

**Affiliations:** 1 Institute for Medical Informatics and Biometry, Faculty of Medicine Carl Gustav Carus, Technische Universität Dresden, 01307 Dresden, Germany; 2 Center for Scalable Data Analytics and Artificial Intelligence (ScaDS.AI), Dresden, Germany

**Keywords:** Health care, Medical research

## Abstract

The emergence of collaborations, which standardize and combine multiple clinical databases across different regions, provide a wealthy source of data, which is fundamental for clinical prediction models, such as patient-level predictions. With the aid of such large data pools, researchers are able to develop clinical prediction models for improved disease classification, risk assessment, and beyond. To fully utilize this potential, Machine Learning (ML) methods are commonly required to process these large amounts of data on disease-specific patient cohorts. As a consequence, the Observational Health Data Sciences and Informatics (OHDSI) collaborative develops a framework to facilitate the application of ML models for these standardized patient datasets by using the Observational Medical Outcomes Partnership (OMOP) common data model (CDM). In this study, we compare the feasibility of current web-based OHDSI approaches, namely ATLAS and “Patient-level Prediction” (PLP), against a native solution (R based) to conduct such ML-based patient-level prediction analyses in OMOP. This will enable potential users to select the most suitable approach for their investigation. Each of the applied ML solutions was individually utilized to solve the same patient-level prediction task. Both approaches went through an exemplary benchmarking analysis to assess the weaknesses and strengths of the PLP R-Package. In this work, the performance of this package was subsequently compared versus the commonly used native R-package called *Machine Learning in R 3* (mlr3), and its sub-packages. The approaches were evaluated on performance, execution time, and ease of model implementation. The results show that the PLP package has shorter execution times, which indicates great scalability, as well as intuitive code implementation, and numerous possibilities for visualization. However, limitations in comparison to native packages were depicted in the implementation of specific ML classifiers (e.g., Lasso), which may result in a decreased performance for real-world prediction problems. The findings here contribute to the overall effort of developing ML-based prediction models on a clinical scale and provide a snapshot for future studies that explicitly aim to develop patient-level prediction models in OMOP CDM.

## Introduction

Prediction models are an essential part of Clinical Decision Support Systems (CDSS) to improve diagnosis, treatment, and risk estimation^1,2^. Machine Learning (ML)-based patient-level prediction models estimate the personal risks and potential outcomes for an individual based on large amounts of data in a time-efficient manner.

To standardize the process of developing prediction models, a prediction framework^3^ has been proposed by the Observational Health Data Sciences and Informatics (OHDSI) Collaborative^4^. OHDSI, which was founded in 2014, develops different computational tools and concepts in the healthcare domain that are compatible with the Observational Medical Outcomes Partnership (OMOP) Common Data Model (CDM)^5^. In particular, the “*Patient-Level Prediction*” (PLP) package that implements the computational framework in R^6,7^, was developed to conduct prediction studies. Here, data analysis and evaluation are possible via standardized data retrieval, annotated pre-processing of data, high-level methods to implement and tune ML models, as well as automatic performance measuring and visualization. To investigate its current strengths and limitations to build ML-based prediction models, we benchmarked the OHDSI tool against the commonly used *Machine Learning in R 3* (mlr3) package^8^, as one prominent example of a native R solution among others (e.g., caret). In particular, we address the following research question in this study: what are the current strengths and weaknesses of the PLP package against more native R based packages, such as mlr3?

To conduct the benchmark, an exemplary clinical prediction model was developed on a synthetic dataset. To achieve a realistic application scenario, an already published use case was recreated to cover the actual clinical aspect of the model. The model is then likewise implemented with PLP and mlr3 in R. For functionalities that are present in mlr3 but not in the PLP package, an additional assessment was made to investigate, if they would be beneficial for the PLP package as well. In the end, an overview of the differences between the packages is provided, i.e., what ML algorithms can be used, how finely they can be tuned, how long the execution time is, the availability of visualization tools, differences in the performance of the models, and the ease of implementation.

## Methods

**Figure 1:**
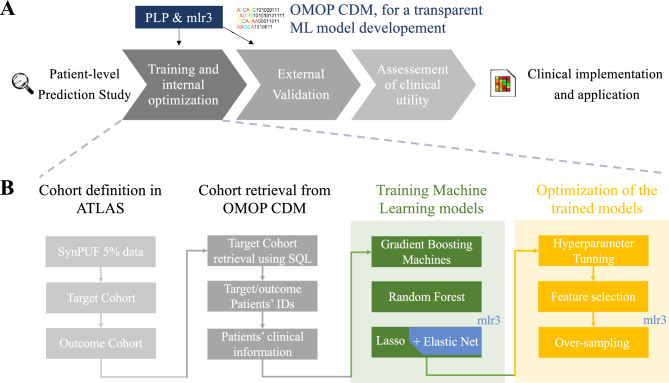
The method pipeline used in the study. (**A**) We trained models using the PLP package from OHDSI community, which is used for design and training of models on OMOP CDM format data and another R community package for design of Machine Learning models, mlr3. In the study a clinical study is recreated. (**B**) The process of ML model training in the study.

Patient-level prediction studies usually contain individual steps for model development, including training and optimization, an external validation of the model by using novel data, as well as the subsequent assessment of the overall clinical utility (Fig. 1A). In our study, we use an OMOP CDM as the basis for our benchmark to compare the PLP and mlr3 packages. In the following, we describe the detailed steps of cohort definition and retrieval, as well as model training and optimization (Fig. 1B).

### Dataset used for building the predictive models

The data source used in this study is the Synthetic Public Use File *"Synpuf 5%"* in the OMOP CDM Version 5.2.2^9^. It contains in total 116,350 synthetic patients and their medical records, which include the various histories of patient features, condition occurrences, treatments, drugs prescribed, measurements, or medical observations. Originally, the data is obtained from real-life patients of a "5% random sample of Medicare beneficiaries in 2008", which were then used to create synthetic patients that are supposed to resemble these patients. The use of synthetic patients enables our investigation to be a sufficiently realistic and transparent use case without requiring any kind of special permission for access unlike for actual patient data. Furthermore, *Synpuf* was chosen because it has a sufficiently large number of patients, was publicly available, and is already present in the OMOP CDM format. The authors confirm that all methods were carried out in accordance with relevant guidelines and regulations with respect to synthetic data.

### Utilized tools

ATLAS^10,11^ is a web application developed by OHDSI, which streamlines the process of creating prediction studies. It provides various features, including cohort definition and prediction study creation over an OMOP database. The tool is user-friendly and requires minimal programming knowledge from users. While a demo version of this web application exists^10^, it is recommended to locally host the web application as this enables the use of custom databases and stores created cohort definitions in a locally created database. The cohorts designed using ATLAS are accessible for use when creating predictive studies with the PLP package. We utilized ATLAS version 2.12.0 in this study.

The PLP Package is the subsequent OHDSI tool to develop ML-based prediction analyses. Here, the package (version 6.3.5) was imported into RStudio version 2023.09 Build 576 (R version 4.3.1). This procedure is described in detail in the handbook of OHDSI^7^.

The mlr3 Package is meant to write custom code with imported functions of the mlr3 Package similar to the PLP tool. mlr3 (Version 0.14.1) was preferred during this study over other available packages, like caret^12^, because of its simplicity and enhanced transparency. In particular, it allows importing all functions necessary for preprocessing, model training, auto feature selection, hyperparameter tuning, evaluation, and visualization.

Since all OHDSI-based tools including PLP are usually written in R, a native R-based package, mlr3, was chosen as the initial step. However, a similar study would also be feasible using an ML-based Python library, such as scikit-learn.

### Cohort definition

To benchmark the two approaches, a patient-level prediction task was needed to evaluate their performances in the entire process of creating prediction models for clinical problems. Therefore, we recreated a prediction study based on the peer-reviewed manuscript from Liu et al., entitled "Prediction of all-cause mortality in coronary artery disease patients with atrial fibrillation based on machine learning models"^13^. In brief, it investigates the relationship between patients with ischemic heart disease who suffer from atrial fibrillation and all-cause death. The paper was selected because it contains patient features that are also present in *Synpuf 5%*, a comparable target cohort size was used, and it also utilized ML models.

Similar to the manuscript from Liu et al.^13^, the entry event for the target cohort consists of the first occurrence of atrial fibrillation for patients with ischemic heart disease. The patient records in *Synpuf 5*% are only available for the years 2008–2010^9^, thus, the time-at-risk was set to three years (2008–2010). The outcome cohort entry event consists of any kind of occurrence of death recorded in the database. The patient features used for the ML models in both mlr3 and PLP approaches in the paper can be grouped into medical conditions (e.g., atrial fibrillation, myocardial infarction, diabetes mellitus, and hypertension), observations (e.g., bleeding history, history of tobacco), drug exposures (e.g., treatment with warfarin, aspirin, beta-blockers), and gender. Therefore, these features, also called covariate groups, were also used in our ML models. The cohort definition was performed using the ATLAS web application and is publicly available for reuse on GitHub^14^. In our PLP approach, parameters, such as “useConditionEraAnyTimePrior = TRUE” as part of the createCovariateSettings() function, ensure that only records before the prediction index are included into the target cohort. In mlr3, we ensured this using a specific filter (condition_era_start_date < cohort_start_date).

The target cohort consists of 18,701 patients, whereas the outcome class consists of 548 patients. For the machine learning classification task, the target population is split into two data frames: 75% training and 25% testing for both PLP and mlr3. Of note, we do not ensure the usage of the same test set for both approaches, however, both implementations employ stratified sampling as a possibility to allow for more balanced ratio between the test and train split for the number of patients used in each approach. This way, we aim to provide a comparable difficulty of the test sets, as in the number of patients with outcomes that need to be detected, is reasonably similar.

### ML study design

#### Data retrieval and pre-processing

Initially, a connection was built to a PostgreSQL server (OMOP database) via the Database-Interface package^15^. Afterwards, patient IDs from the target cohort in the cohort table were extracted and every entry in the observation, drug exposure, person, and condition occurrence that belonged to these patient IDs on the prediction index were extracted and inserted into a data frame in R. The SQL scripts used in this step are available on GitHub as well^14^. The *NA* entries from the obtained tables were replaced by 0 to correct for the absence of a condition or drug exposure and converted other entries to 1 to symbolize the presence of the condition.

#### Model definition

**Figure 2:**
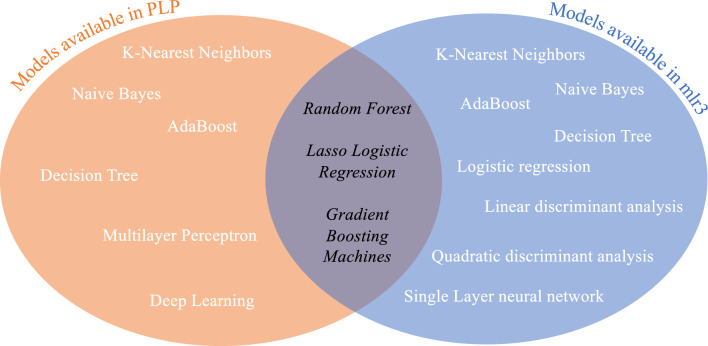
Available models in PLP and mlr3 packages and the utilized models are shown in the middle.

The choice of models in this study was dependent on the availability of the same models in mlr3 and PLP packages and utilized models similar to Liu et al.^13^ to enable utmost comparability. In particular, Liu et al*.* utilize regularization logistic regression, random forest, and support vector machines to conduct their analyses. The list of these available models in both packages and a summary of the finally utilized models are shown in Figure 2. It is worth noting that the models shown in Fig. 2 for the PLP package are the ones that are available by default. The PLP package does support plug-ins of any type of binary classification and custom feature engineering, if users are able to write the custom code.

Of note, mlr3 does not provide a specific Lasso Logistic Regression learner per default, but rather an Elastic Net Regression Learner. Here, Elastic Net combines Lasso and its counterpart Ridge, by setting the hyperparameter *α* to 1, one can achieve pure Lasso^16^. Taken together, the comparison of these three basic classical ML algorithms will serve as a baseline for the overall classification performance.

#### Hyperparameter optimization

PLP uses *grid-search* for hyperparameter optimization without limiting the resolution. Meaning that it calculates a model for every possible combination of hyperparameters values. The values in parentheses are a list of discrete values the hyperparameter can take, e.g., for Random Forest. The utilized hyperparameters for both packages including their tunning intervals are shown in Table 1. The PLP package on the other hand computes 3 ∗ 3 ∗ 1 = 9 different models, each with different hyperparameter settings. The values utilized for PLP models are shown in Table 1 were the default values recommended by OHDSI^17^. We additionally utilized the *scalePosWeight* hyperparameter, which is calculated by dividing the number of patients in the target cohort by the number of patients in the outcome class. This is the inverse value of the ratio of patients with the outcome to the total number of patients. As an illustration, if the incidence ratio is 1 to 100, the scalePosWeight is set to 100. Using this parameter makes misclassifying the positive more costly and therefore trains the model to prioritize resolving these errors. As a result, disadvantages of imbalanced data can be minimized in some cases.

**Table 1:** Hyperparameters tuned for each model in both PLP and mlr3 packages and their tuning intervals.

Model	Hyperparameter	PLP	mlr3
Random forest	*max depth*	17	–
	*mtries*	$\sqrt{nFeatures}$	–
	*ntrees*	(500, 750, 1000, 1250, 1500, 2000)	(500–1000)
Lasso logistic regression	*Starting Variance*	0.1	–
	*s*	–	(− 12 to 12)
Gradient boosting machines	*earlyStopRound*	25	25
	*scalePosWeight*	40	40
	*learningRate*	(0.001, 0.005, 0.01, 0.05, 0.1)	(0.0001–1)
	*ntrees*	1000	-

On the other hand, in mlr3 the learners were wrapped by a hyper-parameter auto-tuner. For each learner, the hyperparameter space has been manually defined. For each model, this space indicates hyperparameters that should be tuned within particular intervals. A hyperparameter that is not utilized in PLP is the parameter *s* for the Lasso Logistic Regression Model. This hyperparameter controls the *λ* hyperparameter, which in turn controls the *shrinkage* inside the Lasso Regression Function^18^. The PLP Lasso Logistic regression implementation performs an automatic tuning for regularization^19^. Further colloquial s or λ tuning can be done using the Cyclops Package^20^, which implements the Lasso Logistic Model in the PLP package.

The hyperparameter spaces for mlr3 were initially selected following Bischl et al*.*^21^ but then modified for this study. Due to time constraints and limited computational power, the number of hyperparameter configurations tested for each training run was set to 5. Therefore, the used hyperparameters have been reduced to 1 to match the computational budget. For the Gradient Boosting Machine model, the *scalePosWeight* was also set to a constant number and the *earlyStopRounds* number has been taken from the PLP Model. The search method was set to random to ensure time efficiency. The number of hyperparameter combinations to test was set to 5.

#### Auto-feature selection

In addition to an auto-tuning algorithm, an auto-feature selection algorithm selects the significant covariates that improve the utility evaluation metrics. Since Elastic Net already had an inbuilt feature selection in the form of the LASSO penalty function applied to the Regression^18^, no external feature selection was used. Therefore, the auto-feature selection was only implemented for the GradientBoostingMachine and Random Forest.

#### Additional oversampling techniques

The mlr3 Package supports SMOTE^22^, an oversampling strategy, which was applied and evaluated on the Lasso Logistic Regression Model. Additionally, the trained hyperparameters and their interval for the SMOTE are shown in Table 2.

**Table 2:** Hyperparameter space definitions for oversampling (SMOTE) methods.

Sampling method	Function	Parameter in PLP	Parameter in mlr3
*SMOTE*	smote.k	–	1–6
	smote.dup_size	–	1–6

#### Comparison and performance metrics

Since we are mimicking a clinical study during this work, it is more important to detect a patient with an actual outcome rather than mislabeling a non-outcome patient. In ML, it is defined as minimizing the false negatives, therefore increasing the recall of the model.

In general, our dataset is imbalanced as is common in clinical studies^23^. In other words, the number of instances in the outcome cohort is rare compared to those in the non-outcome cohort the 2.36% of the population. In absolute numbers, there are 18,701 patients in the target cohort overall, and from those 548 patients died within the time-at-risk. Notably, this has an influence on the selection of the evaluation metrics, as stated by Japkowicz^24^. For instance, if a model classifies all patients in the target cohort as the majority cohort, in this case, the non-outcome cohort, while the minority cohort only exists in 2% of the target population, then the model has reached an accuracy of 98%. Therefore, the choice of metric here is important. Taking this aspect into account, Precision and Recall, and the Area-Under-The-Curve (AUC) of the Precision-Recall curve (PRC), which combines Recall and Precision, were used as performance measurement metrics with a 95% Confidence Interval (CI)^24^. Additionally, we measured the model calibration via calculating the Brier Score for each model^25^.

### Computational infrastructure

Our computations were executed on a 32 GB RAM machine with a 2,6 GHz 6-Core Intel Core i7.

## Results

We used the *Synpuf 5%* dataset and defined our cohort using the ATLAS web application. The included cohort in the analysis included 18,701 Patients. We trained Random Forest, Lasso Regression, and Gradient Boosting Machines in both PLP and mlr3 packages with default parameters. Elastic Net Regression is utilized as the substitute model for Lasso Logistic Regression mlr3.

### Comparison of model performances between mlr3 and PLP

Out of all models available in PLP and mlr3, Gradient Boosting Machines (GBM) performed best for this prediction problem at least in terms of AUC (ROC) score. As shown in Table 3, the Elastic Net Model that is not supported as default in the PLP package, however, the performance is slightly decreased in comparison to GBM for AUC (ROC). However, some models performed close to the performance of a classifier randomly guessing, in other words of a random classifier.

**Table 3:** The AUC (ROC), AUC (PRC), and brier scores for all models.

Model	PLP			mlr3		
	AUC (ROC)	AUC (PRC)	Brier score	AUC (ROC)	AUC (PRC)	Brier score
Random forest	0.56 [0.53, 0.59]	0.029 [0.029, 0.029]	0.16 [0.16, 0.17]	0.50 [0.50–0.50]	0.495 [0.494, 0.495]	0.02 [0.02, 0.02]
Lasso logistic regression	0.53 [0.50, 0.56]	0.026 [0.025, 0.027]	0.02 [0.02, 0.02]	**0.88 [0.87, 0.89]**	0.500 [0.500, 0.500]	0.01 [0.01, 0.01]
Gradient boosting machines	0.54 [0.52, 0.57]	0.029 [0.028, 0.030]	0.15 [0.13, 0.17]	0.51 [0.50, 0.51]	0.660 [0.659, 0.661]	0.25 [0.25, 0.25]
Elastic net logistic regression	–	–	–	0.87 [0.86, 0.88]	**0.871 [0.856, 0.885]**	0.01 [0.01, 0.01]

The metrics for PLP and mlr3 models are calculated with a 95% Confidence Interval after ten iterations. Confidence Interval values for each metric are represented in: metric [lower bound, upper bound].

Significant values are in bold.

### Execution time

No formal timing has been performed to measure the execution time of these packages. However, start time and end time have been logged and the differences between these times can be seen in Table 4.

**Table 4:** Execution times of the different models (h = hour, min = minutes, and sec = seconds).

Model	PLP	mlr3
Random forest	17 min 30 sec	47 min 27 sec
Lasso logistic regression	1 min 30 sec	8 min 21 sec
Gradient boosting machines	2 min 45 sec	24 min
Elastic net logistic regression	–	8 min 19 sec

### Results with SMOTE applied

We exemplarily applied SMOTE oversampling to the best-performing model based on AUC (ROC) in mlr3. The results are shown in Table 5 along with the baseline experiments before oversampling were applied. Our results indicate a decrease in AUC (ROC) and AUC (PRC) after the oversampling method was applied.

**Table 5:** The performance of the lasso logistic regression model in both packages in the baseline experiment and after applying the SMOTE method.

	Model	Baseline		SMOTE	
		AUC (ROC)	AUC (PRC)	AUC (ROC)	AUC (PRC)
mlr3	Lasso logistic regression	0.88	0.500	0.589	0.034

## Discussion

Our best-performing models in mlr3 achieved reasonable AUC (ROC) and AUC (PRC) scores, that are on average comparable to the models used by Liu et al.^13^. One of the reasons for the relatively low performance of some of our models in comparison to the best performing models of Liu et al. (i.e., Random Forest) could be the high dimensionality of the covariates, in other words, the large number of features used. In our study, we had 10,940 whereas Liu et al. used only 58 features. Even though auto-feature selection was used, a greater computational budget would have been required to correctly sort out any noise features that distort the performance of the models. Another factor could be that there is simply only a low correlation between ischemic heart disease with atrial fibrillation and all-cause death in the synthetic dataset used here. No external validation was performed by Liu et al., therefore, the performance of their trained models on different populations, such as the population created from the *Synpuf 5%* dataset is unclear. A third factor could be the usage of synthetic data as such, which might not be a clear representative of real-world data.

However, since our primary aim was not to create the best possible prediction models but to compare the overall model calculation between the packages, a weak model performance should not impact the ability to compare these packages greatly.

### Model performances

The results show that there are differences between the regression models of the mlr3 implementation and the rest of the tested models. The reason the regression models perform better overall could be attributed to the fact that this model is better suited for this prediction problem^13^. The Lasso Logistic Regression also performed better than Random Forest and Gradient Boosting Machines for AUC (ROC). Interestingly, the Lasso Logistic Models differ greatly between the mlr3 package and the PLP package. The reason for this difference could be that the hyperparameter optimization in PLP might be not extensive enough, at least in the Lasso Logistic Model. Another indicator would be that the implementation of the Lasso Logistic Model in the mlr3 package outperforms the one in the PLP package. It is worth mentioning that without hyperparameter tuning in the mlr3-Lasso’s performance drops to about the same level as the model in the PLP implementation. In this study, our primary aim was to compare the default hyperparameters for PLP for a baseline comparison. The hyperparameter values for both approaches were tuned to ensure comparability. We strived to maintain the hyperparameters and the tuning values as similar as possible in both PLP and mlr3 approaches.

### Execution time favors the usage of the web-based PLP application

As indicated in the results, the PLP package has a rather shorter execution time overall as shown in Table 4. The reason for this could be the integrated packages in the PLP packages. For example, the Cyclops package is designed for the "Regression of very large problems" for "up to millions of observations, millions of variables"^20^. However, as PLP uses grid search for hyperparameter optimization, execution times increase exponentially for every new hyperparameter, which can lead to longer execution times if multiple hyperparameters need to be optimized. mlr3-based models could also perform faster, but modeling and optimizing them would take some time and effort. In comparison, PLP package models are already time-optimized.

### Performance of SMOTE indicates no difference

The effects of applying SMOTE is shown in Table 5. Here, it indicates that applying SMOTE has a negative impact on the performance of the model. More models and optimally on a real-world dataset need to be tested with and SMOTE to finally depict any kind of performance increases, as was already shown in related studies, such as Bej et al.^26^.

### Comparison of the ease of implementation

Our study showed that the design of computational models in PLP was easier and faster than in the mlr3 package. In particular, the PLP package essentially streamlines data retrieval and pre-processing. Of course, the PLP package is meant to operate on OMOP-based data and therefore the data structure was clear for the developers of the package. Therefore, the way the data must be extracted from the data source and how it needs to be restructured is already known and only has to be implemented once. Nonetheless, it is crucial to bear in mind that the PLP package is continually evolving, and there may be instances where certain functions become non-operational when transitioning between versions, particularly while updating to the latest releases. For example, features like visualization can temporarily become inaccessible, necessitating collaboration with the developers, as was the case during our initial testing phase with version 6.0.4 of the package (see details here: https://github.com/OHDSI/PatientLevelPrediction/issues/337). While this issue posed a significant challenge for our research, it is imperative to recognize that encountering such obstacles is a common and expected occurrence when dealing with packages in active development. Anticipating and addressing these challenges is a fundamental aspect of working with evolving software packages. In contrast in mlr3, it is the responsibility of the user to adjust the data retrieval and preprocessing steps. While the mlr3 package could be utilized in different problems, the PLP package presents high-level functions for every step because the data structure is already known. This includes all processing phases between data retrieval pre-processing, as well as classification, in which underlying technical details are already sorted out. Therefore, these functions only needed to be called in settings, so that the user can focus more on the clinical application rather than the technical implementation.

Additionally, the PLP package offers a directed visualization option that can be quite useful for sharing and interpreting the outcomes by creating a plot e.g., for the number of covariates used, ROC and PRC Curves, sensitivity, and specificity. No directly implemented equivalent was found in the mlr3 package and its sub-package *mlr3viz*, but R offers other suitable packages that could be utilized for this purpose, such as RShiny^27^.

### Who should prefer mlr3 over PLP?

The PLP package is designed for OMOP-based data, which can be quite useful and time-efficient for performing studies within the OHDSI community and visualizing the results using already built-in functions on ATLAS and other OHDSI tools. Additionally, since PLP is designed as an application, no extensive programming knowledge is required to use it. The PLP approach includes current ML classifiers and can handle large data sets in a reasonable time, as PLP, once correctly configured, automatically retrieves all features and stores them in an SQLite database, and thus saves RAM capacity.

The backend of many of the binary classifiers in the PLP package is scikit-learn^28^, which might be an additional reason for PLP’s faster performance. PLP additionally, includes informative visualizations.

If users seek to implement more explicit and particular ML-classifiers or other related approaches, such as oversampling, mlr3 or other native R-packages offer larger freedom to operate. However, the computational knowledge needs to be more extensive, since many processing steps have to be done manually (e.g., pre-processing, normalization, classification, and visualization). In particular, if data is stored in the OMOP format, the model built in the mlr3 package needs to be adjusted accordingly. Moreover, performing extensive analysis using machine learning on a large cohort, such as ours (18,701*10,941), on a machine with small storage can lead to longer processing times. Therefore, a smarter data storage solution is required to ensure scalability. Which is why we additionally utilized the mlr3db package^29^ to improve the running time for our mlr3 models. Thus, due to our current investigation and snapshot of both approaches, it would be a trade-off between high flexibility (mlr3) and ease of applicability (PLP).

## Conclusion

In this work, the goal was to assess the strengths and weaknesses of the PLP package in conducting patient-level prediction models. For that, two separate approaches were developed, one using imported functions from the PLP package and one using imported functions from the mlr3 package and its sub-packages.

It was shown that the developed models most of the time performed similarly in their prediction performance. However, weaknesses were detected in the default implementation of the Lasso Logistic Regression in the PLP package in comparison to the Lasso Logistic Regression Model implemented with mlr3.

Nonetheless, there was a noticeable difference observed in the execution time between the packages. The PLP package performed exceptionally well and computed its models quicker than the mlr3 package. This is a first indicator of scalability as it shows it can handle larger amounts of data in a time-efficient manner. Additionally, it was found that the PLP package has a wide variety of time-efficient tools to visualize the models' results. Another advantage of the PLP package is its ease of implementation compared to mlr3 since data retrieval is streamlined in the PLP package.

Taken together, the strengths and weaknesses indicated during this study for both packages give a current overview for any future studies aimed to develop patient-level prediction models in OMOP CDM. Both approaches can compute ML-based patient-level prediction models on OMOP CDM and might be selected due to the user background and application scenario.

## Data Availability

The script used for analysis in this article is available on GitHub^14^. The *Synpuf* dataset is available on^9,30^.
